# Supplementary material for: Yiai Fuzheng decoction inhibits triple-negative breast cancer by remodeling the immune microenvironment
Source: Front Immunol. 2025 Sep 30;16:1615631. doi: 10.3389/fimmu.2025.1615631 (PMC12518410; doi:10.3389/fimmu.2025.1615631)
Supplement: Supplementary file 2 [file DataSheet1.zip › Supplementary File 2/Treatment/C_vs_D_vs_H/06_Pathway_Analysis/Pathway_Analysis/Sig_Pathways_Name/Pentose and glucuronate interconversions.pdf]

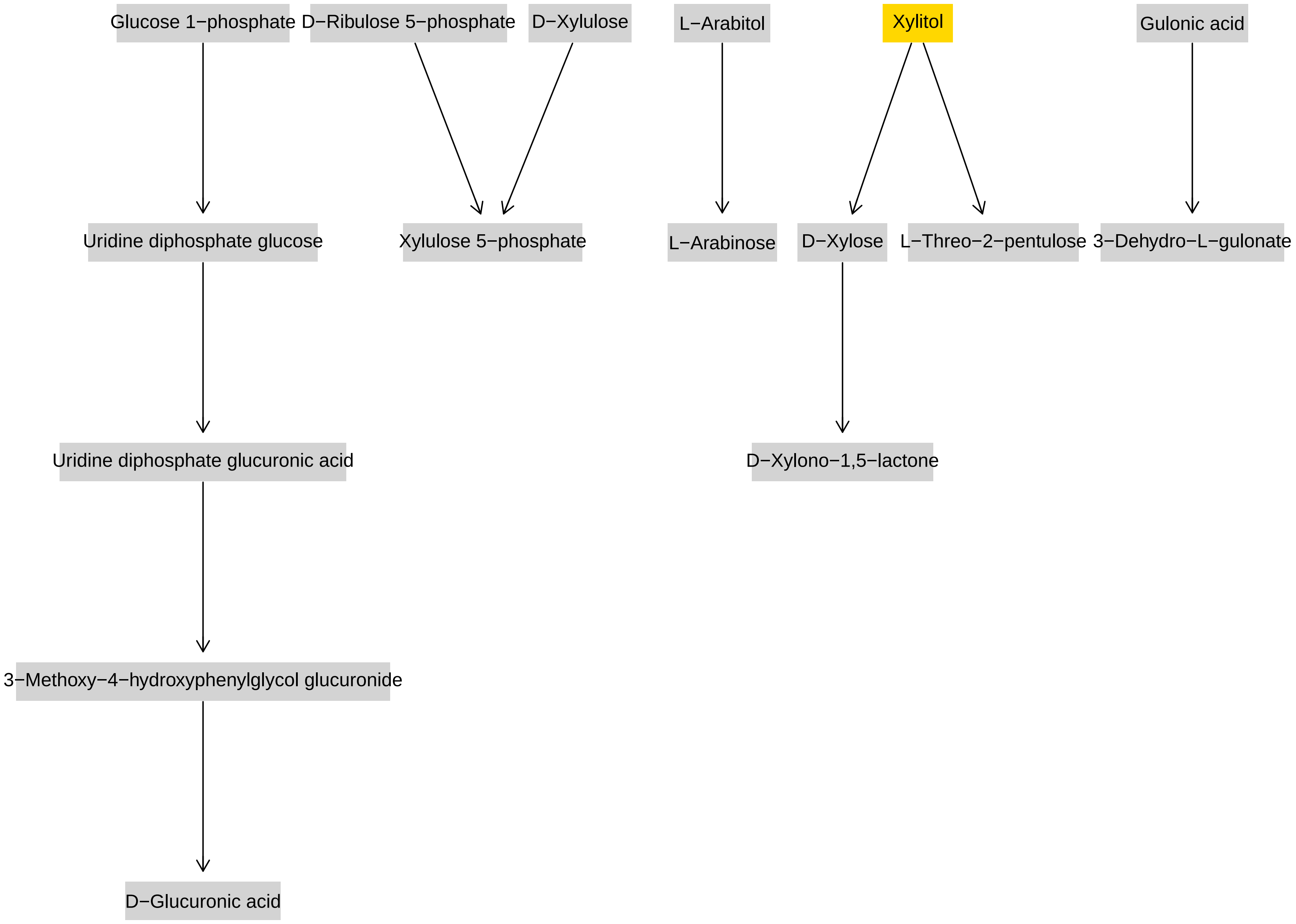

Glucose 1−phosphate
D−Ribulose 5−phosphate
D−Xylulose
L−Arabitol
Xylitol
Gulonic acid
Uridine diphosphate glucose
Xylulose 5−phosphate
L−Arabinose
D−Xylose
L−Threo−2−pentulose
3−Dehydro−L−gulonate
Uridine diphosphate glucuronic acid
D−Xylono−1,5−lactone
3−Methoxy−4−hydroxyphenylglycol glucuronide
D−Glucuronic acid
